# Effective soil erosion control represents a significant net carbon sequestration

**DOI:** 10.1038/s41598-018-30497-4

**Published:** 2018-08-13

**Authors:** Lishan Ran, Xixi Lu, Nufang Fang, Xiankun Yang

**Affiliations:** 1 Department of Geography, The University of Hong Kong, Pokfulam Road, Pokfulam, Hong Kong; 2 Department of Geography, National University of Singapore, Singapore, Singapore; 3 College of Ecology and Environment, The University of Inner Mongolia, Hohhot, China; 4 State Key Laboratory of Soil Erosion and Dryland Farming on the Loess Plateau, Institute of Soil and Water Conservation, Northwest A&F University, Yangling, Shaanxi Province China; 5 School of Geographical Sciences, Guangzhou University, Guangzhou, China

## Abstract

The debate over whether soil erosion is a carbon (C) sink or atmospheric CO_2_ source remains highly controversial. For the first time, we report the magnitude of C stabilization associated with soil erosion control for an entire large river basin. The soil erosion of the Yellow River basin in northern China is among the most severe worldwide. Progressive soil conservation has been implemented by the Chinese government since the 1970s, including the largest ever revegetation programme, the Grain-for-Green Project, which began in 1999. Based on compiled hydrological records and organic carbon (OC) data, together with primary production estimates, we evaluated the sequestered OC resulting from soil conservation. Compared with that at baseline in 1950–1970, in which significant soil conservation did not occur, the fate of erosion-induced OC was substantially altered in the period from 2000–2015. Approximately 20.6 Tg of OC were effectively controlled per year by soil conservation efforts. Simultaneously, the decomposition of erosion-induced soil organic carbon (SOC) declined from 8 Tg C yr^−1^ to current 5.3 Tg C yr^−1^. The reduced C emissions (2.7 Tg C yr^−1^) within the Yellow River basin alone account for 12.7% of the mean C accumulation acquired via forest expansion throughout all of China previously assessed. If the accumulated C in restored plants and soils was included, then 9.7 Tg C yr^−1^ was reduced from the atmospheric C pool during this period, which represents a tremendous C-capturing benefit. Thus, the increased C storage obtained via soil conservation should be considered in future C inventories.

## Introduction

Soil erosion is one of the most challenging environmental problems facing human society, and it has garnered widespread attention worldwide because of the associated land degradation, which is closely correlated with human livelihoods^1–3^. In addition to the physical consequences, such as crop yield reductions and sedimentation, soil erosion has recently been shown to represent a significant biogeochemical factor for the carbon (C) cycle. The accelerated agricultural soil erosion caused by extensive land use over recent decades has been found to represent a C sink of 60–270 teragrams of carbon per year (Tg C yr^−1^)^4,5^, although contrasting findings have also been reported^6,7^. Great uncertainty is inherent in erosion-induced C flux changes, which remain to be properly addressed^8^. Nevertheless, erosion-induced soil organic carbon (SOC) has become an important C pathway in the global C cycle. Tentative estimates of SOC flux and its fate during deposition have been incorporated into recent C budget assessments^9−11^. Despite the recognition that erosion-induced SOC is subject to various human disturbances, few quantitative evaluations have been performed on the changes in erosion-induced SOC over time. The recent renewed awareness of the global significance of soil erosion and conservation in climate change mitigation has further illustrated the urgency of performing systematic investigations into SOC dynamics^11,12^. Focusing only on hillslope erosion sites or sub-catchments isolated from land-ocean connectivity would result in diverse and even contradictory findings. Thus, the dynamics of SOC within a complete river system, from the upland areas to the ocean, must be assessed to provide critical insights into the different pathways of SOC on a landscape scale and determine the potential impacts of human disturbances.

**Figure 1:**
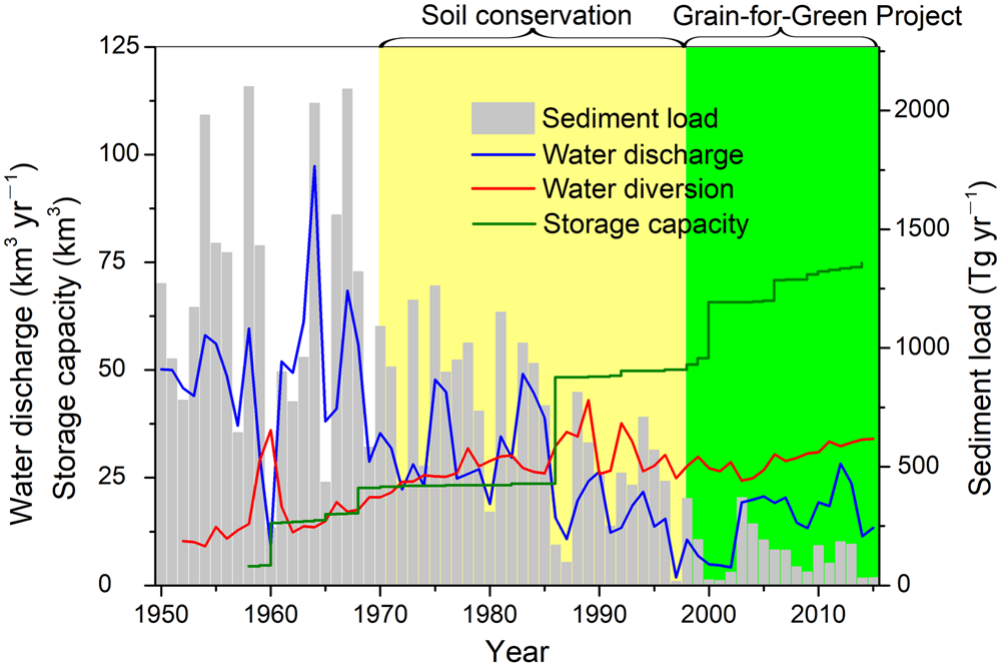
Temporal variations of the Yellow River basin in the reservoir storage capacity, water diversion, and water and sediment fluxes into the Bohai Sea from 1950–2015.

The Yellow River basin (Supplementary Fig. S1) is home to a vibrant population of 140 million people, and it has the highest soil erosion rate worldwide because of a combination of unique soil properties, the hydrological regime, and extensive human pressure^13,14^. To mitigate soil erosion and improve land productivity, progressive soil conservation along with other engineering interventions, such as the construction of reservoirs and check dams, have been widely implemented in the Yellow River basin since the 1970s (Fig. 1). The Grain-for-Green Project, which is the largest-ever ecological restoration programme in human history, was initiated in 1999 and presented the ambitious goals of restoring the degraded ecosystem services and combatting erosion by converting cropland to forest and grassland. Carefully selected tree, shrub, and grass species adaptable to the local arid climate have been planted on former croplands. Consequently, both the soil erosion rate and sediment flux have been substantially reduced (Fig. 1), and the delivery of ecosystem services has been improved accordingly^15,16^. The present sediment flux into the ocean accounts for only 10% of that in the 1950s (ref.^17^). Profound changes in soil erosion and subsequent sediment transport have inevitably modified the cycling processes of C, which is closely associated with erosion and sediment dynamics. Furthermore, after more than 10 years of vegetation restoration, the once severely deteriorated ecological environment has been significantly restored^18,19^, although massive efforts are still needed.

Compared with climate change, human activities are considered the major causes of reduced soil erosion and sediment flux^14,20,21^. Although the impact on water and sediment dynamics has been widely investigated, holistic assessments of the associated C capture remain largely unknown. A lack of robust estimates of C stabilization represents one of the greatest challenges for combating climate change^22,23^. Thus, we aimed to quantitatively evaluate the amount of C controlled by human activities accompanying soil erosion control and ecological restoration within the Yellow River basin. We investigated human-induced C capture by comparing the organic carbon (OC) budget of two scenarios: a baseline from 1950–1970 before the implementation of large-scale soil conservation practices and a scenario from 2000–2015 after the introduction of the Grain-for-Green Project (Methods).

## Results

### Increases in net primary production (NPP) and SOC stock

**Figure 2:**
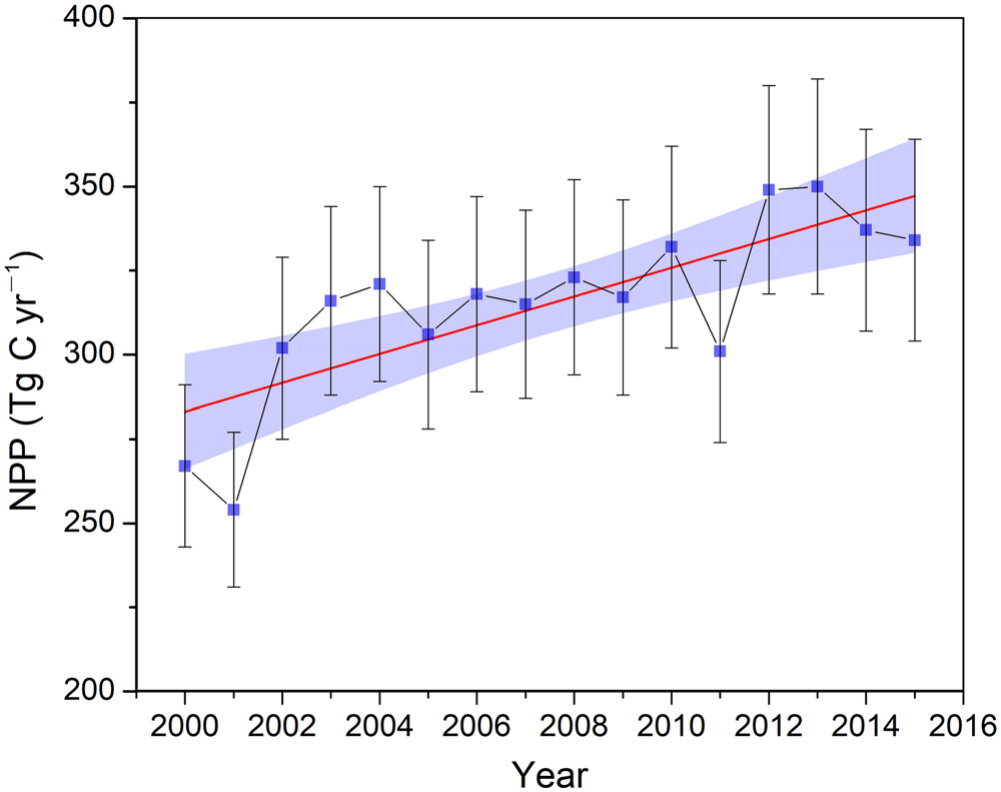
Steadily increasing NPP within the Yellow River basin from 2000–2015. The red straight line denotes a linear regression between NPP and the year: *y* = 4.27*x* − 8258 (r^2^ = 0.59; *p* < 0.001), and the shaded area represents the 95% confidence intervals of the linear fitting. Error bars denote standard deviation.

Since the implementation of the Green-for-Grain Project in 1999, the vegetation coverage on the Loess Plateau has nearly doubled from 31.6% in 1999 to 59.6% in 2013 (ref.^24^). The improved ecosystem within the Yellow River basin has increased the terrestrial OC storage in both the biomass and soil horizons because of the enhanced C inputs^15,25^. We first examined the temporal trends in annual net primary production (NPP) (Supplementary), which exhibited a steady increase over the period 2000–2015 despite inter-annual variability and considerable uncertainty associated with annual estimations (Fig. 2). The annual NPP increased from 267 ± 24 Tg C in 2000 to 334 ± 30 Tg C in 2015 with an average incremental rate of ~4.3 Tg C yr^−1^ (Fig. 2). When normalized to the drainage area of the Yellow River basin, the NPP was 355 ± 32 g C m^−2^ yr^−1^ in 2000 and 444 ± 40 g C m^−2^ yr^−1^ in 2015 (Supplementary Table S1), thus representing a 25% increase.

In addition to the fixed OC by biome, the SOC stock have simultaneously increased. The top-soil layers in the restored woodland and grassland areas exhibited the highest increases in SOC content, whereas the SOC stock in the subsoil layers (>40 cm deep) expanded slowly. After cropland conversion and abandonment, reforestation and grass restoration exhibited different SOC accumulation rates. Grassland can generally store 20–75% more SOC than woodland, and the magnitude of SOC sequestration in a woodland is highly dependent on the maturity and species of the planted trees^26,27^. Based on 1366 soil profiles collected from the Loess Plateau (Supplementary Fig. S3), we estimated the impact of vegetation restoration (i.e., the Grain-for-Green Project) on the accumulation of SOC stock on the Loess Plateau and in the Yellow River basin. Compared with the initial cropland with a relatively lower SOC stock, the restored woodland and grassland areas collectively sequestered SOC at a rate of 55 ± 18 g C m^−2^ yr^−1^ in the top 0–100 cm of soil. Accordingly, the SOC stock accumulation rate within the Yellow River basin was conservatively estimated at 2.7 ± 0.9 Tg C yr^−1^.

### Temporal changes in erosion-induced SOC

**Figure 3:**
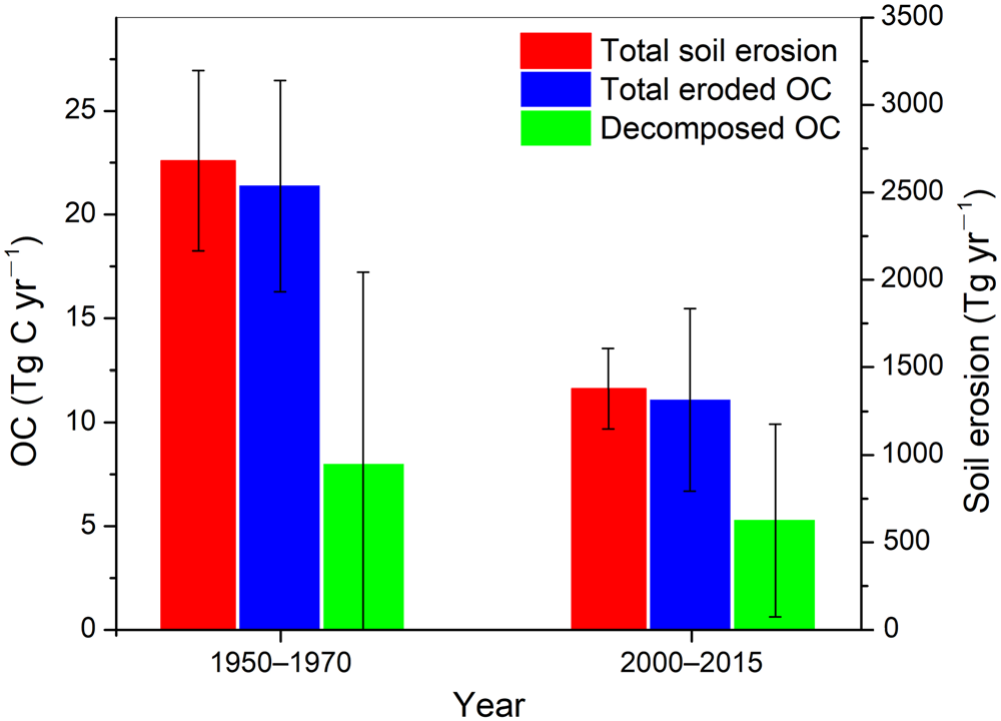
Significant reductions in soil erosion and OC mobilization after large-scale human interventions within the Yellow River basin. The range bars represent the associated uncertainties.

During the baseline scenario, in which effective measures had not been introduced to control soil erosion and trap fluvial sediments, the total eroded soil in the Yellow River basin was estimated at 2680 Tg yr^−1^ (Fig. 3). This value corresponds to a mean soil erosion rate of approximately 7000 t km^−2^ per year on the Loess Plateau, which supplies nearly 90% of the Yellow River’s sediment^28,29^. Approximately 1200 Tg of sediment was discharged annually into the ocean during this period (Supplementary Table S3). The remaining 1480 Tg was deposited on land, mainly in the lower main-stem channel. As a result of large-scale human interventions, including soil conservation on hillslopes, dams constructed in valleys and along river channels, and vegetation rehabilitation across the watershed and on the Loess Plateau in particular, soil erosion intensity has been greatly reduced in recent decades. Compared with that in the baseline scenario, the total erosion under the 2000–2015 scenario decreased by 52% to 1380 Tg yr^−1^ (Fig. 3), which amounts to a mean soil erosion rate of only 3000 t km^−2^ per year on the Loess Plateau. Thus, less eroded sediment has reached the ocean and more has been deposited on land (Supplementary Table S3).

With respect to the OC cycle, the mobilized SOC in the baseline scenario was 21.4 ± 5.2 Tg C yr^−1^ on average (Fig. 3). In comparison, the OC flux delivered into the Bohai Sea was 6.1 ± 4.3 Tg C yr^−1^, and the amount deposited with sediments through fluvial sedimentation, especially in the lower Yellow River main-stem (mean sediment deposition rate: 420 Tg yr^−1^), was 7.3 ± 5.7 Tg C yr^−1^ (Supplementary Table S3). This indicates that only 28.5% of the eroded SOC was eventually transported further downstream into the ocean. Based on the budgetary equation, approximately 8 Tg of SOC was lost per year during transit largely due to decomposition (Fig. 3), which is substantially higher than the horizontal seaward flux and accounts for 37.4% of the eroded SOC. In the last 16 years, the amount of mobilized SOC decreased to 11.1 Tg C yr^−1^ because of soil conservation and vegetation restoration, thus representing 52% of that in the baseline scenario (Fig. 3 and Supplementary Table S3). Simultaneously, the decomposed SOC decreased to 5.3 Tg C yr^−1^, suggesting a 34% decline from the baseline scenario (Fig. 3).

**Figure 4:**
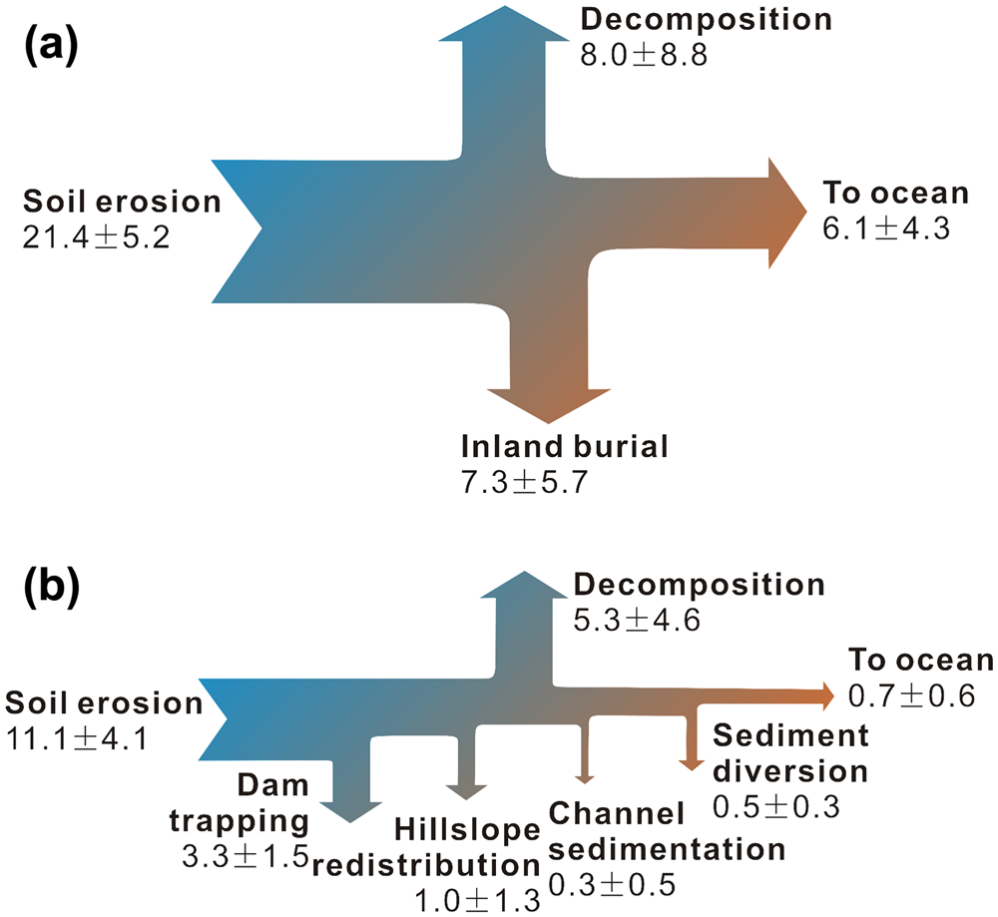
Impact of progressive soil conservation on the erosion-induced SOC dynamics in the Yellow River basin (in units of Tg C yr^−1^). Panel (a): baseline period from 1950–1970; Panel (b): period from 2000–2015 after the introduction of the Grain-for-Green Project. The line widths of the arrows in each panel are approximately proportional to the SOC fluxes.

Compared with the baseline scenario, approximately 10.3 Tg C was reduced per year as a result of hillslope soil conservation (21.4 Tg C yr^−1^ versus 11.1 Tg C yr^−1^; Fig. 4). With an average sediment deposition rate of 940 Tg yr^−1^ (Supplementary Table S3), another 3.3 ± 1.5 Tg C per year was buried behind dams, which includes approximately 3100 reservoirs and 110,000 silt check dams^30,31^. Although water withdrawal from the main-stem channel, mainly for agriculture, has increased steadily over this timeframe (Fig. 1), the diverted OC is relatively minimal (0.5 ± 0.3 Tg C yr^−1^; Supplementary Table S3). Owing to the vulnerability of the diverted C to further decomposition by tillage practices, this flux was not accounted for when evaluating the total C capture.

The differences in SOC flux between the two scenarios reveal the effect of soil conservation (Fig. 4). Particularly, its impact on the Yellow River’s basin-scale OC cycle can be quantitatively assessed from two perspectives: the accumulated NPP and SOC stock acquired through rehabilitated plants and the OC captured via sediments because of the implementation of soil conservation measures. Summing up the two terms suggests that soil conservation aimed at soil erosion control collectively reduced 20.6 Tg C yr^−1^ from 2000–2015. Moreover, the reduced C emissions (2.7 Tg C yr^−1^) accounted for 63% of the NPP accumulation (4.3 Tg C yr^−1^).

## Discussion

### Uncertainty analysis of the budgets

One of the key sources of uncertainty in this assessment of SOC dynamics is the rate of soil erosion (see the detailed uncertainty analysis for each budget term in Supplementary). Because human-induced C control is based on a budgetary analysis, its significance and accuracy in modifying the basin-scale OC cycle relies on the amount of eroded SOC. The soil erosion value used in this evaluation was based on a sediment delivery ratio (SDR) of 0.9, and this value is believed to reflect the high sediment delivery characteristics of the Yellow River and represents a substantially higher level than other large rivers, which generally have a much lower SDR of 0.1–0.3 (ref.^32^). If a lower SDR was adopted, both the eroded SOC and the decomposed OC would considerably increase (Supplementary Fig. S10). For example, the mobilized SOC may be 38.5 Tg C yr^−1^ under the baseline scenario if the SDR was set to 0.5, and, accordingly, the decomposed OC would almost double to 14.6 Tg C yr^−1^. In this regard, the decomposed fraction would be much higher than the sequestered OC in sediments, resulting in a huge C source into the atmosphere. Despite the uncertainty of the individual budget terms (Supplementary Table S3), a comparative analysis of the two scenarios indicates that aggressive human interventions aimed primarily at controlling soil erosion have played a positive role in capturing C.

Another potential uncertainty is likely associated with the estimate of SOC stock changes resulting from the implementation of the Grain-for-Green Project. The mean annual SOC stock accumulation of 2.7 Tg C was based on the land use change from cropland to woodland and grassland by assuming a lower SOC stock in the reference cropland. This may have probably overestimated the actual incremental rate of SOC stock generated directly by soil conservation practices, because the cropland itself has stored huge quantities of SOC. Extensive use of mulches, conservation tillage, degradation of crop residues, and application of chemical fertilizers and manures in cropland have greatly enhanced SOC stock on the Loess Plateau^33,34^. Furthermore, the estimation was based solely on land use conversion from cropland (mostly terraced cropland). This is not necessarily true as other land use types, such as apple and jujube orchards, were also abandoned for vegetation restoration, but with a significantly lower magnitude. These land cover types generally exhibited reference SOC contents different from that in cropland^35,36^. In comparison with the reference cropland, recent studies suggest that the SOC content of top soils in orchard land could be ~30% higher^37^, but slightly lower SOC contents are also reported^38^. Considering the small spatial extent of orchard land abandonment, however, its impact on estimation of total SOC stock accumulation within the entire Yellow River basin should be minimal. Our annual SOC stock accumulation result is consistent with the recent estimate of 1.7–2.9 Tg C yr^−1^ for the Loess Plateau^27^.

### Implications for erosion-induced C assessments

The direct reduction of SOC mobilization caused by decreased soil erosion is primarily the result of soil conservation on hillslopes (Fig. 4). Vast areas of gentle slope lands (<25°) were converted into terraces to decrease surface runoff velocity and preserve soils. The proportion of terrace farming on the Loess Plateau has steadily expanded from 2% in 1979 to 9% in 2006 (ref.^21^). Furthermore, vegetation restoration has also been pursued since the 1970s, although to a much smaller extent than that under the Grain-for-Green Project. For example, vegetation coverage on the Loess Plateau increased by 4–6.6% by 1999 (refs ^21,24^). These measures have effectively protected hillslope soils from being swept away, thereby fixing SOC on the uplands. As an important strategy to mitigate erosion, soil conservation on hillslopes has generated significant C stabilization co-benefits by increasing the on-site soil C pool and reducing the SOC losses. Moreover, although the amount of fixed C from recently restored ecosystems remains small, it will likely further increase with the continuous expansion of revegetation from cropland conversions as suggested by Fig. 2.

A long-running debate remains over the contribution of erosion-induced SOC dynamics towards curbing climate change^4,6^. Although few studies based on field or local-scale monitoring on the Loess Plateau demonstrated that C replacement at eroding sites can fully replenish the mobilized C and leads erosion process to be a C sink^39,40^, it is worth noting that these studies are mostly based on agricultural systems with intensive fertilizer use. Erosion in the Yellow River basin as a whole has been recognized as a net C source into the atmosphere^29^. This can also be confirmed by SOC storage changes over the 30-year interval (i.e., the period 1970–1999). The Loess Plateau ecosystem had been a net C source due to SOC loss by erosion until 2000 and actually, the NPP balance in 2000 was still negative (i.e., −11 Tg C) despite the initiation of the Green-for-Grain Project^15^. Another concern regarding the impact of soil conservation is to what extent it could affect the basin-wide OC storage dynamics. The riverine sediment OC in the Yellow River is biogeochemically refractory^41^ and is only 36–39% lower than the eroded SOC contents for both scenarios (Supplementary Table S3). This suggests that a considerable fraction (i.e., 61–64%) of the eroded SOC may not be degraded during transit, but instead re-deposited somewhere within the river system or on the Bohai seafloor. Even so, considering the high magnitude of soil erosion and SOC mobilization, it appears that aggressive soil conservation is obviously necessary and beneficial. That is, more eroded SOC would have been decomposed or discharged into the Bohai Sea and less would have been sequestered in the river system if soil conservation measures had not been conducted.

Compared with land-use changes (i.e., deforestation) that emit greenhouse gases into the atmosphere, these soil control measures capture considerable amounts of C that have rarely been accounted for in traditional C-cycling studies^11,42^. This C control is expected to be more substantial at larger spatial scales in which depositional processes dominate the transport and re-distribution of the eroded SOC. Of the controlled 20.6 Tg C yr^−1^, soil conservation measures implemented on hillslopes and sediment trapping behind dams are the most effective strategies, and they jointly fix 13.6 Tg C yr^−1^. In comparison, the fixed C from increased NPP and SOC stock is relatively small (~7 Tg C yr^−1^), which is largely because of the arid climate, scarce water availability, and immaturity of recently planted trees^43^. Although engineering practices, such as dams and terracing, can respond quickly to C sequestration upon completion, a lag effect is observed for the restored plants, which typically take several years to maximize C-fixation efficiency (Fig. 2).

From the perspective of mitigating atmospheric CO_2_ increase, approximately 9.7 Tg C was reduced every year by soil conservation that would otherwise be emitted into the atmospheric C pool. This flux includes the C fixation in restored plants and soils and the reduced C emissions. It is biogeochemically significant and can affect land-atmospheric C exchange within the watershed. The average CO_2_ emissions in China caused by fossil fuel burning and cement production were 1.42 t C yr^−1^ per capita from 2000–2014 (ref.^44^). When applied to the Yellow River basin, the total C emissions from the two sources were 198 Tg C yr^−1^. Therefore, the C removed from the atmosphere compensated for 5% of these emissions, and the total C control through soil conservation represented approximately 10.4% of the emissions. Particularly, the direct reduction in C emissions (2.7 Tg C yr^−1^) within the Yellow River basin alone accounts for 12.7% of the mean C accumulation acquired via forest expansion throughout all of China^45^. These percentages will further increase with further soil conservation and land management efforts. For example, more than 160,000 silt check dams will be completed in the coming years on the Loess Plateau^31^. Our synthesis demonstrates that soil conservation projects are not only directly effective for mitigating erosion but are also promising for capturing C.

In tropical and temperate ecosystems with high *in situ* C replacement, soil erosion tends to be beneficial for the removal of CO_2_ from the atmosphere over long timescales^32,46^; therefore, this approach should be endorsed to some extent. However, for arid ecosystems, such as the Yellow River basin, our results indicate that soil conservation is essential. Aggressive soil erosion control, although not implemented for the purpose of C sequestration, led to a significant reduction in C mobilization and release. Conservation practices and not erosion processes constitute a real C sequestration. Nevertheless, although soil conservation has been conducted over a long time period worldwide, quantitative assessments of the resulting changes to SOC dynamics and their implications for terrestrial ecosystems remain limited over large spatial scales^8^. Sustainable land management by reducing soil erosion carries notable climate benefits for erosion-related C assessments. Thus, recognizing and understanding the magnitude of this conservation-induced C storage dynamics is crucial. It is of global importance to incorporate soil conservation into SOC mobilization and terrestrial C cycling processes. Resolving these issues will not only reduce current uncertainties in C budget estimates but will also facilitate the implementation of effective mitigation and adaptation strategies in response to global climate change.

## Methods

### Study area

The Yellow River originates from the Qinghai-Tibetan Plateau at an elevation of 4000–6000 m and flows eastward through the Loess Plateau and then along the North China Plain, ultimately emptying into the Bohai Sea (Supplementary Fig. S1). The drainage area is 752,000 km^2^, and the basin is located in an arid-semiarid climate with a mean annual temperature of 8–14 °C in most parts. Precipitation in the basin is low and highly spatially uneven, and it decreases from 700 mm yr^−1^ in the southeast to 250 mm yr^−1^ in the northwest^29^. Due to the strong soil erosion and high sediment yields, the Yellow River was once categorized as having the largest sediment flux, and it transported 1080 Tg of sediment into the ocean per year over the 66-year period from 1950–2015 (refs^17,21^). Severe sedimentation within channel has caused a unique geographical landscape of ‘hanging river’ in the upper and lower Yellow River main-stem with the riverbed 3–10 m higher than the surrounding ground^47,48^.

The Loess Plateau covers an area of approximately 385,000 km^2^, mainly within the middle reaches of the Yellow River (Supplementary Fig. S1), and is a major source of sediment, although it provides only 44% of the water as measured at the Huayuankou gauge station^14^. Although the headwater landscape is largely covered by alpine meadow, the Loess Plateau is sparsely vegetated and covered mainly by grassland ecosystems. Since the implementation of the Grain-for-Green Project in 1999, the area of forest and grassland has steadily increased. By 2008, woodland had increased by 4.9% and shrubland and grassland had increased by 6.6%, whereas cultivated cropland decreased by 10.8% over the same period^15^. More detailed descriptions are available in the Supplementary.

### Calculation of the annual NPP

Ecosystem NPP defines the amount of atmospheric CO_2_ fixed by plants through photosynthesis that is accumulated as biomass. To calculate the annual ecosystem NPP in the Yellow River basin, we used version-55 of the Terra/MODIS NPP products (MOD 17A3) produced by the Numerical Terradynamic Simulation Group (NTSG)/University of Montana (UMT) (https://lpdaac.usgs.gov/dataset_discovery). The annual NPP was produced at a 1-km spatial resolution based on MODIS remote-sensing data with a temporal coverage from 2000–2015. The accuracy of the annual NPP in version-55 was estimated within 9%; thus, it was ready for use^49^. Within the Yellow River basin boundary, the annual NPP from 2000–2015 was calculated, and the results are presented in Supplementary Table S1.

### Accumulation of SOC stock

To evaluate the impact of revegetation on SOC stock, we compiled 1366 soil profiles of different land use types from 21 studies in the literature (Supplementary and Table S2). By analysing the SOC density differences of these soil profiles under different land use types, we estimated the resulting accumulation of SOC stock after the implementation of the Grain-for-Green Project.

### OC budget and uncertainty analyses

We analysed the production, transport, and deposition of SOC induced by erosion through established sediment and OC budget equations (Supplementary). In the baseline scenario (1950–1970), significant human interventions had not been conducted to reduce soil erosion in the basin. The eroded soils from hillslopes present two main destinations: natural deposition on land and transport into the ocean. With this simple budget equation, the decomposed OC was estimated. In the 2000–2015 scenario, major human impacts on sediment and OC transport dynamics were identified. These impacts included slope soil conservation, dam trapping, and water diversion from the main-stem channel.

The sediment cycle (subscript: *S*) is described as follows:

1 $${E}_{S}={T}_{S}+{H}_{S}+{W}_{S}+{O}_{S}+{R}_{S}$$

where *E*, *T*, and *H* represent eroded soils, dam trapping, and channel deposition, respectively; and *W, O*, and *R* represent water diversion, seaward transport, and hillslope redistribution, respectively.

The OC cycle (subscript: *C*) is described as follows:

2 $${E}_{C}={T}_{C}+{H}_{C}+{W}_{C}+{O}_{C}+{R}_{C}+{D}_{C}$$

The additional flux (*D*_*C*_) represents the decomposed OC during fluvial transport in the river system. *D*_*C*_ is calculated as a residual between the eroded and deposited OC. Detailed descriptions of the budgets can be found in Ran *et al*. (ref.^29^).

We first analysed the uncertainty of each quantifiable budget term (Supplementary and Table S3). Because *R*_*S*_ and *D*_*C*_ were identified as residuals among the eroded, deposited, and transported quantities, we assessed their propagation of uncertainty by treating the uncertainty in the individual terms as statistically independent, although this was not entirely true^7,29^.

## Electronic supplementary material

Supplementary Information

## Data Availability

The budgetary results of OC transport and NPP estimates for the Yellow River basin are available in the Supplementary or from the corresponding author upon request.

## References

[1] Montgomery DR (2007). Soil erosion and agricultural sustainability. Proceedings of the National Academy of Sciences.

[2] Pimentel D (2006). Soil erosion: a food and environmental threat. Environment, development and sustainability.

[3] García-Ruiz JM (2015). A meta-analysis of soil erosion rates across the world. Geomorphology.

[4] Van Oost K (2007). The impact of agricultural soil erosion on the global carbon cycle. Science.

[5] Harden JW (2008). Soil erosion: data say C sink. Science.

[6] Lal R, Pimentel D (2008). Soil erosion: a carbon sink or source?. Science.

[7] Smith SV, Renwick WH, Buddemeier RW, Crossland CJ (2001). Budgets of soil erosion and deposition for sediments and sedimentary organic carbon across the conterminous United States. Global Biogeochemical Cycles.

[8] Borrelli P (2017). An assessment of the global impact of 21st century land use change on soil erosion. Nature communications.

[9] Battin TJ (2009). The boundless carbon cycle. Nat Geosci.

[10] Wehrli B (2013). Biogeochemistry: Conduits of the carbon cycle. Nature.

[11] Chappell A, Baldock J, Sanderman J (2015). The global significance of omitting soil erosion from soil organic carbon cycling schemes. Nature Climate Change.

[12] Chabbi A (2017). Aligning agriculture and climate policy. Nature Climate Change.

[13] Chen J (2005). Spatial and temporal analysis of water chemistry records (1958–2000) in the Huanghe (Yellow River) basin. Global Biogeochemical Cycles.

[14] Wang HJ (2007). Stepwise decreases of the Huanghe (Yellow River) sediment load (1950–2005): Impacts of climate change and human activities. Global and Planetary Change.

[15] Feng, X., Fu, B., Lu, N., Zeng, Y. & Wu, B. How ecological restoration alters ecosystem services: an analysis of carbon sequestration in China’s Loess Plateau. *Scientific Reports* **3**, 10.1038/srep02846 (2013). 10.1038/srep02846 PMC3789145 24088871

[16] Sun W (2015). Spatiotemporal vegetation cover variations associated with climate change and ecological restoration in the Loess Plateau. Agricultural and Forest Meteorology.

[17] Yellow River Conservancy Commission. Yellow River Sediment Bulletin, (http://www.yellowriver.gov.cn/nishagonggao/). Access: 25 April 2016 (2016).

[18] Xin ZB, Xu JX, Zheng W (2008). Spatiotemporal variations of vegetation cover on the Chinese Loess Plateau (1981–2006): Impacts of climate changes and human activities. Sci China Ser D.

[19] Zhang B, Wu P, Zhao X (2011). Detecting and analysis of spatial and temporal variation of vegetation cover in the Loess Plateau during 1982–2009. Transactions of the Chinese Society of Agricultural Engineering.

[20] Peng J, Chen S, Dong P (2010). Temporal variation of sediment load in the Yellow River basin, China, and its impacts on the lower reaches and the river delta. Catena.

[21] Wang S (2015). Reduced sediment transport in the Yellow River due to anthropogenic changes. Nat Geosci.

[22] Mayorga E (2008). Carbon cycle: Harvest of the century. Nature.

[23] Lal R (2004). Soil carbon sequestration impacts on global climate change and food security. Science.

[24] Chen Y (2015). Balancing green and grain trade. Nat Geosci.

[25] Wang Y, Fu B, Lü Y, Chen L (2011). Effects of vegetation restoration on soil organic carbon sequestration at multiple scales in semi-arid Loess Plateau, China. Catena.

[26] Deng L, Wang G, Liu G, Shangguan Z (2016). Effects of age and land-use changes on soil carbon and nitrogen sequestrations following cropland abandonment on the Loess Plateau, China. Ecological Engineering.

[27] Zhao B (2017). Spatial distribution of soil organic carbon and its influencing factors under the condition of ecological construction in a hilly-gully watershed of the Loess Plateau, China. Geoderma.

[28] Hassan MA, Church M, Xu J, Yan Y (2008). Spatial and temporal variation of sediment yield in the landscape: Example of Huanghe (Yellow River). Geophys Res Lett.

[29] Ran L, Lu XX, Xin ZB (2014). Erosion-induced massive organic carbon burial and carbon emission in the Yellow River basin, China. Biogeosciences.

[30] Ran L, Lu XX, Xin ZB, Yang X (2013). Cumulative sediment trapping by reservoirs in large river basins: A case study of the Yellow River basin. Global and Planetary Change.

[31] Zhang H (2016). Loess Plateau check dams can potentially sequester eroded soil organic carbon. Journal of Geophysical Research: Biogeosciences.

[32] Doetterl S (2016). Erosion, deposition and soil carbon: A review of process-level controls, experimental tools and models to address C cycling in dynamic landscapes. Earth-Science Reviews.

[33] Liu Z, Shao MA, Wang Y (2011). Effect of environmental factors on regional soil organic carbon stocks across the Loess Plateau region, China. Agriculture, Ecosystems & Environment.

[34] Zhang C, Liu G, Xue S, Sun C (2013). Soil organic carbon and total nitrogen storage as affected by land use in a small watershed of the Loess Plateau, China. Eur J Soil Biol.

[35] Wang Z (2017). Soil organic carbon on the fragmented Chinese Loess Plateau: Combining effects of vegetation types and topographic positions. Soil and Tillage Research.

[36] Gao X, Meng T, Zhao X (2017). Variations of soil organic carbon following land use change on deep‐loess hillsopes in China. Land Degradation & Development.

[37] Wang Y, Fu B, Lü Y, Song C, Luan Y (2010). Local-scale spatial variability of soil organic carbon and its stock in the hilly area of the Loess Plateau, China. Quaternary Research.

[38] Wang R (2018). Contrasting responses of soil respiration and temperature sensitivity to land use types: Cropland vs. apple orchard on the Chinese Loess Plateau. Sci Total Environ.

[39] Zhao J, Oost KV, Chen L, Govers G (2016). Moderate topsoil erosion rates constrain the magnitude of the erosion-induced carbon sink and agricultural productivity losses on the Chinese Loess Plateau. Biogeosciences.

[40] Li Y (2015). Sustained high magnitude erosional forcing generates an organic carbon sink: Test and implications in the Loess Plateau, China. Earth and Planetary Science Letters.

[41] Wang X, Ma H, Li R, Song Z, Wu J (2012). Seasonal fluxes and source variation of organic carbon transported by two major Chinese Rivers: The Yellow River and Changjiang (Yangtze) River. Global Biogeochemical Cycles.

[42] Yue Y (2016). Lateral transport of soil carbon and land− atmosphere CO_2_ flux induced by water erosion in China. Proceedings of the National Academy of Sciences.

[43] Chen L, Wei W, Fu B, Lü Y (2007). Soil and water conservation on the Loess Plateau in China: review and perspective. Progress in Physical Geography.

[44] World Bank. World Development Indicators. http://data.worldbank.org/indicator/EN.ATM.CO2E.PC?locations=CN (Carbon Dioxide Information Analysis Center, Environmental Sciences Division, Oak Ridge NationalLaboratory, Tennessee, United States, 2017).

[45] Fang J, Chen A, Peng C, Zhao S, Ci L (2001). Changes in forest biomass carbon storage in China between 1949 and 1998. Science.

[46] Wang Z (2017). Human-induced erosion has offset one-third of carbon emissions from land cover change. Nature Climate Change.

[47] Pan B (2015). Sediment grain-size characteristics and its source implication in the Ningxia–Inner Mongolia sections on the upper reaches of the Yellow River. Geomorphology.

[48] Yu Y (2013). New discharge regime of the Huanghe (Yellow River): causes and implications. Continental Shelf Research.

[49] NASA LP DAAC. Terra/MODIS Net Primary Production Yearly L4 Global 1 km https://lpdaac.usgs.gov/dataset_discovery/modis/modis_products_table/mod17a13 Access: 18 August 2016 (2016).

