# Supplementary material for: Effective soil erosion control represents a significant net carbon sequestration
Source: Sci Rep. 2018 Aug 13;8:12018. doi: 10.1038/s41598-018-30497-4 (PMC6089926; doi:10.1038/s41598-018-30497-4)
Supplement: Supplementary file 1 — Supplementary Information [file 41598_2018_30497_MOESM1_ESM.pdf]

Supplementary for

Effective soil erosion control represents a significant net carbon sequestration

Lishan Ran[a *], Xixi Lu[b,c *], Nufang Fang[d] and Xiankun Yang[e]

[a] Department of Geography, The University of Hong Kong, Pokfulam Road, Hong Kong
[b] Department of Geography, National University of Singapore, Singapore
[c] College of Ecology and Environment, University of Inner Mongolia, Hohhot, China
[d] State Key Laboratory of Soil Erosion and Dryland Farming on the Loess Plateau, Institute of Soil and Water Conservation, Northwest A&F University, Yangling, Shaanxi, China
[e] School of Geographical Sciences, Guangzhou University, Guangzhou, China
* *Correspondence*: lsran@hku.hk and geoluxx@nus.edu.sg

This file includes

1. **Location of the Yellow River basin**
2. **Carbon (C) fixation by vegetation restoration**
3. **Assessment of soil erosion**
4. **Assessment of SOC dynamics**
5. **Fluxes into the Bohai Sea**
6. **Uncertainty with SDR-based estimates**
7. **Presentation of OC budgetary results**

1. Location of the Yellow River basin

As the second longest river in China and the six longest in the world, the Yellow River (Huang He in Chinese) originates on the eastern edge of the Qinghai-Tibetan Plateau (Figure S1). Its total river length is 5464 km. With a drainage area of around 752,000 km^2, it flows eastward through the Loess Plateau and then along the North China Plain, before finally discharging into the Bohai Sea (Figure S1). From the geological perspective, the catchment above Lanzhou gauge station is situated on the uplifting Qinghai-Tibetan Plateau, whereas the middle reaches overlap largely with the Loess Plateau and the lower reaches greatly confined on the submerging North China Plain. The Loess Plateau, composed of highly erodible loess layers of 30–200 m thick, is the major source of the Yellow River sediment while providing ~40% of water discharge [1,2].

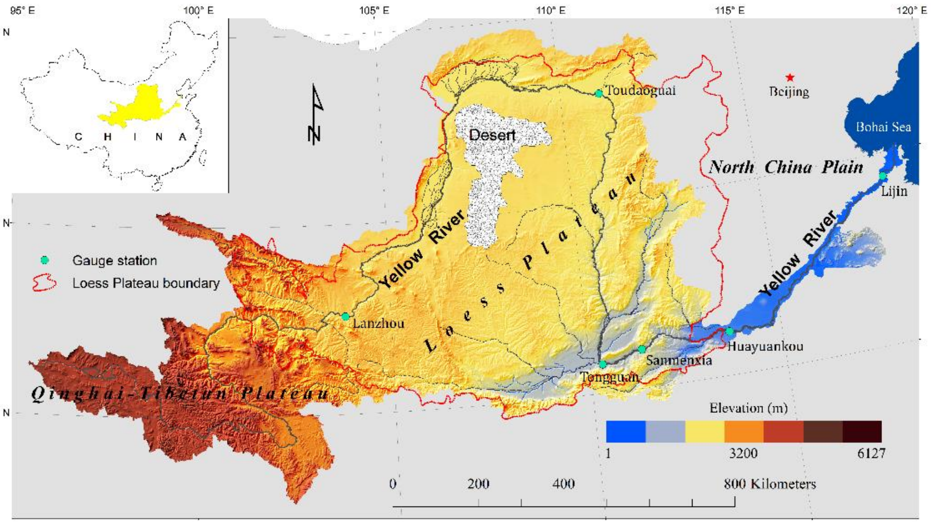

Figure S1. Location map of the Yellow River basin overlapping with the highly erodible Loess Plateau in its middle reaches. Sediment fluxes at Sanmenxia gauge station are generally used to represent the total sediment load from the Loess Plateau [3].

Precipitation in the basin is characterized by pronounced seasonal fluctuations. Approximately, 85% of the precipitation falls during the wet months from June to September with the remaining 15% occurring in the dry season [4]. Affected by low precipitation, the multi-year average surface runoff across the watershed is 77 mm only [5]. Consequently, the natural water discharge not significantly affected by human activities, such as dam storage and water withdrawals, was estimated to be 58 km^3 per year [6]. However, the water discharge into the Bohai Sea has showed a stepwise decline in recent decades as a result of large-scale human impacts. Present water discharge is 18 km^3 per year. Concomitant reductions have also been observed in sediment and other materials transported with water or sediment by the same order of magnitude [4,7].

2. Carbon (C) fixation by vegetation restoration

We first estimated the fixed atmospheric CO_2 by restored plants within the Yellow River basin from two perspectives: the net primary production (NPP) and the soil organic carbon (SOC) stock. For NPP, we used version-55 of the Terra/MODIS NPP products (MOD 17A3). These products are produced by the Numerical Terradynamic Simulation Group (NTSG)/University of Montana (UMT) and downloaded from https://lpdaac.usgs.gov/dataset_discovery. The annual NPP was produced at a 1-km spatial resolution based on MODIS remote sensing data with a temporal coverage from 2000 through 2015 for the Yellow River basin. Based on the data quality

report attached with the data set, the accuracy of the annual NPP products was assessed within 9% and thus was ready for research purpose [8]. The accumulated NPP within the Yellow River basin boundary increased steadily during the period from 2000 to 2015 (Figure S2). If the year 2000 was assumed to represent the reference, the recovering vegetation coverage can be clearly detected across the whole watershed, on the middle and northern Loess Plateau in particular. Accordingly, the number of grids with low NPP values have been shrinking over the period, particularly on the northern Loess Plateau with exceptionally low precipitation. The calculated annual NPP accumulation rate is presented in Table S1.

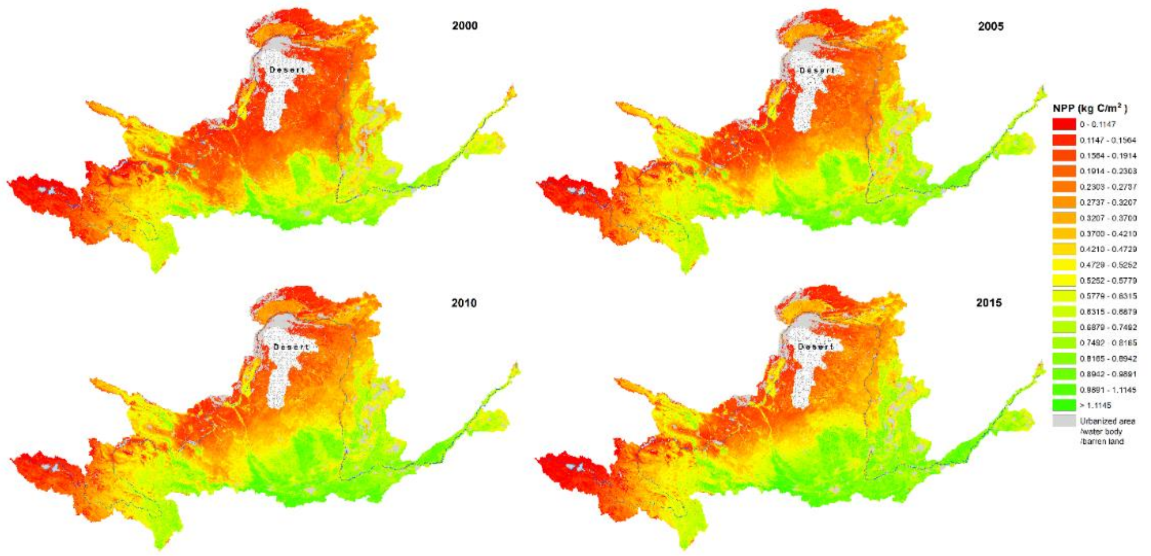

Figure S2. Temporal evolution of the annual NPP within the Yellow River basin during the period 2000–2015 after the implementation of the Grain-for-Green Project.

Table S1. Annual NPP changes in the Yellow River basin during the period 2000–2015 (expressed as mean ± standard deviation).

Year	NPP (Tg C)	NPP (g C m^{-2})
2000	267±24	355±32
2001	254±23	337±30
2002	302±27	402±36
2003	316±28	420±38
2004	321±29	427±38
2005	306±28	406±37
2006	318±29	422±38
2007	315±28	419±38
2008	323±29	430±39
2009	317±29	421±38
2010	332±30	442±40
2011	301±27	400±36
2012	349±31	465±42
2013	305±32	466±42
2014	337±30	448±40
2015	334±30	444±40

Vegetation restoration can increase not only C storage within biome, but also the SOC stock within soil. Extensive implementation of the Grain-for-Green Project and previous vegetation restoration programmes since the 1970s have improved SOC storage dynamics [9,10]. Numerous studies based on the Loess Plateau have explicitly demonstrated that its SOC density has been continuously increasing and the amount of total SOC stock has also been expanding. To assess the resulting changes to SOC stock, we compiled 1366 soil profiles from 21 studies widely distributed on the Loess Plateau (Figure S3 and Table S2). Analyzing the SOC density changes along soil profiles under different land use types indicates that the top 0–40 cm soil layers exhibited the highest increase in SOC while the deeper soils (>40 cm) showed much lower SOC stock changes [11-14]. After cropland conversion and abandonment, the grassland areas can generally store 20–75% more SOC than the woodland areas, and the SOC sequestration rates in woodland areas are highly dependent on the maturity and species of the planted trees [10,13-15].

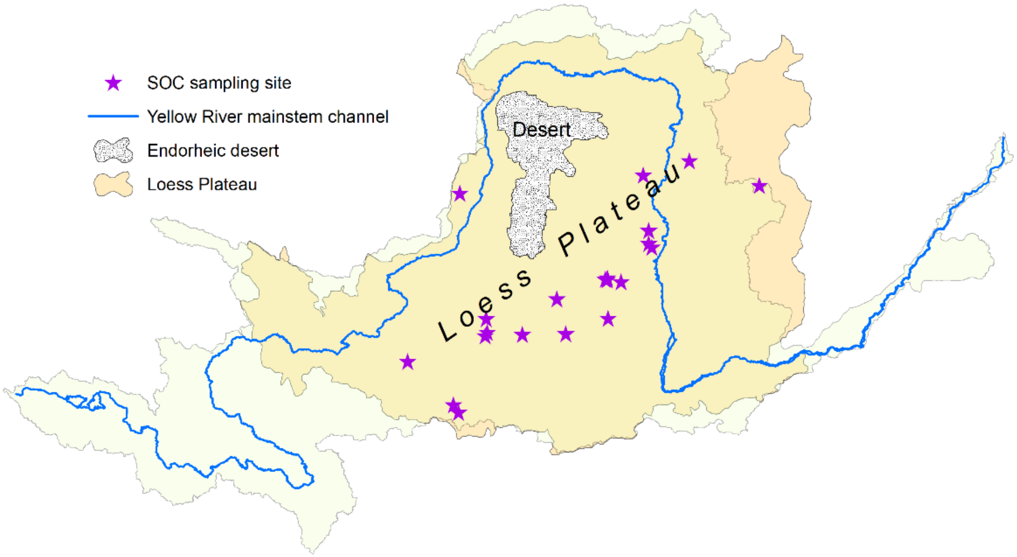

Figure S3. Sampling locations of the compiled SOC studies on the Grain-for-Green Project on the Loess Plateau. The retrieved soil profiles from the literature are presented in Table S2.

A simplified synthesis of the compiled soil profiles indicates that, compared with the initial cropland, the restored woodland and grassland collectively sequestered SOC at a rate of 55±18 g C m^{-2} yr^{-1} for the top 0–100 cm soils. This rate is comparable to recent meta-analysis of SOC accumulation rate of 59 g C m^{-2} yr^{-1} on the Loess Plateau and across China [16]. Because the area of restored vegetation has been continuously expanding from 2000–2015, we used the total area of vegetation restoration by the year of 2008, the middle of the study period, to estimate the incremental rate of SOC stock resulting from vegetation restoration. By 2008, approximately 4.83 Mha of cropland on the Loess Plateau had been converted to grassland and woodland [17]. The SOC stock within the Yellow River basin were conservatively increasing at a rate of 2.7±0.9 Tg C yr^{-1} over the recent decade. In comparison, the soil sampling results collected on the middle Loess Plateau by Zhao et al. (2017) [14] suggest that the net increase in its SOC stock due to the implementation of the Grain-for-Green Project is 1.7–2.9 Tg C yr^{-1}. Apparently, our estimate is consistent with this sampling result. This suggests that our SOC stock accumulation rate estimate can be used for assessment with medium-to-high confidence. The restored plants, including grassland and woodland, play an important role in increasing the SOC stock.

Table S2. Compiled soil profiles for the SOC accumulation analysis within the Yellow River basin.

Catchment	Location	Coordinate		Area	Temperature	Rainfall	Number of soil profiles	Reference
		E	N	km^2	^{o}C	mm/yr		
Anjiapo	Gansu	104.65	35.5833	5.65	N.D.	427	56	Chen et al., 2007 (ref. 11)
Liudaogou	Shaanxi	110.3583	38.5833	P.S.	7-9	437	48	Fu et al., 2010 (ref. 18)
Yangjuangou	Shaanxi	109.5833	36.7	2.02	N.D.	535	105	Wang et al., 2010 (ref. 19)
Zhifanggou	Shaanxi	109.2294	36.7783	P.S.	8.8	505	129	Wang et al., 2010 (ref. 20)
Chenghuang	Shanxi	113	38.1	P.S.	10.7	555	18	Chen et al., 2009 (ref. 21)
Luoyugou	Gansu	105.6122	34.7391	72	N.D.	534	180	Xin et al., 2016 (ref. 13)
Zhifanggou	Shaanxi	109.2667	36.7667	8.27	8.8	510	68	Zhao et al., 2016 (ref. 22)
Ziwuling	Shannxi	109.201	36.067	P.S.	9	577	22	Chang et al., 2012 (ref. 23)
Lianjiabian	Gansu	108.2224	35.8636	P.S.	10	587	15	Deng et al., 2013 (ref. 24)
Nanxiaohe	Gansu	107.2528	35.9256	1.92	9.3	557	28	Jin et al., 2014 (ref. 25)
Shuanghuang	Guyuan	106.4667	36.0167	P.S.	7	400	99	Cheng et al., 2015 (ref. 26)
Yunwushan	Ningxia	106.4667	36.2667	N.D.	6.9	400-450	16	Chang et al., 2017 (ref. 12)
Ansai	Shaanxi	109.2789	36.7958	P.S.	9.1	503	95	Deng et al., 2016 (ref. 15)
Wangmaogou	Shaanxi	110.3406	37.5703	5.97	10	513	216	Zhao et al., 2017 (ref. 14)
Qiaozi	Gansu	105.7167	34.6	1.36	10.7	542.5	27	Li et al., 2017 (ref. 27)
Anshai	Shaanxi	105.0956	36.5125	P.S.	8.8	505.3	60	Han et al., 2012 (ref. 28)
Sidigou	Ningxia	106.4131	35.9567	5.16	6.9	419	46	Fang et al., 2012 (ref. 29)
Yuanzegou	Shaanxi	110.3620	37.2620	0.58	8.6	505	37	Zhao et al., 2015 (ref. 30)
Helanshan	Ningxia	106.0667	38.5833	P.S.	8.5	200	15	Wu et al., 2013 (ref. 31)
Badaogou	Shanxi	110.3	37.3333	P.S.	4	830.8	72	Zhao et al., 2010 (ref. 32)
Wuzhai	Shanxi	111.4667	38.7333	P.S.	4.1	400	14	Wang et al., 2007 (ref. 33)

Note: P.S. denotes plot scale studies and corresponding information on catchment area is not available, and N.D. represents no data.

3. Assessment of soil erosion

We estimated the soil erosion in the baseline 1950–1970 scenario based on field-based measurements of sediment load at hydrological gauge stations within the Yellow River basin. There are 225 gauge stations operated by the Yellow River Conservancy Commission (Figure S4), which is the official agency that supervises the hydrology of the Yellow River and is affiliated to the Ministry of Water Resources of China. A selection criteria of at least 5-year-long sediment records was set to filter out gauge stations deemed not representative of soil erosion and sediment yielding, due largely to hydrological cycling (i.e., extremely high or low rainfall). Furthermore, some gauge stations that are on main-stem or major tributary channels received sediment from various geomorphological, hydrological, and geological settings. The measured sediment at these gauge stations have probably obscured the signature of actual sediment yielding and transport processes within their respective upstream catchment. As a result, 35 gauge stations were manually removed from analysis. In addition, another 11 gauge stations located shortly downstream of reservoirs, despite only a few reservoir constructions during this period, were also discarded because of the greatly reduced sediment load. Finally, 179 gauge stations were retained for spatial interpolation estimation of sediment yielding and soil erosion amount. The catchment area that these gauge stations control varied greatly in scale from 19 km^2 to more than 10,000 km^2. Out of the 179 catchments, 42% of which are less than 1000 km^2. This

suggests that the sediment measurements at most of the gauge stations used are representative of sediment yielding on hillslopes and gullies.

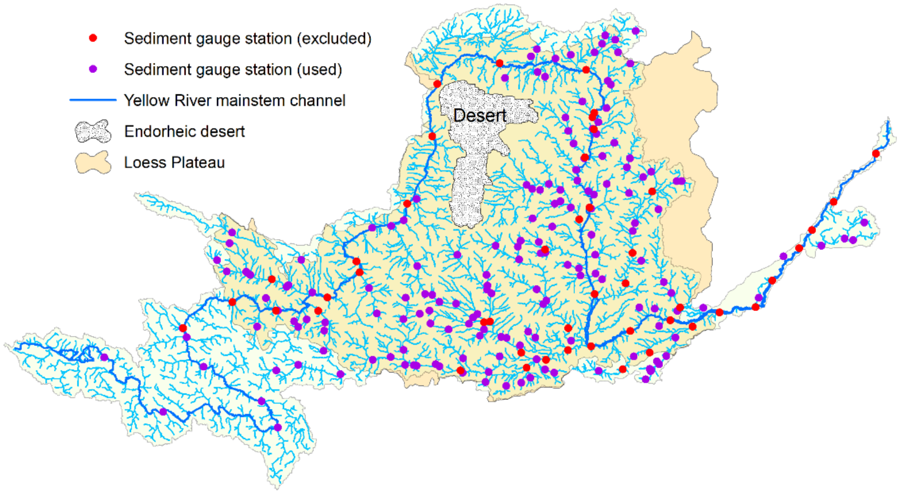

Figure S4. Locations of the sediment gauge stations within the Yellow River basin. Also shown is the Loess Plateau that largely overlaps with the middle Yellow River basin.

We employed the Kriging interpolation method to estimate basin-wide sediment yielding [2]. The result is presented in Figure S5 and has been reported in our earlier work [34]. The sediment yield within the Yellow River basin is characterized by pronounced spatial variability. While extremely high values (i.e., >20,000 t km^{-2} yr^{-1}) were commonly observed on the Loess Plateau, the upper and lower Yellow River basin exhibited low sediment yields due to relatively better vegetation cover and less human perturbations (Figures S4 and S5). Based on the generated sediment yield map, we further estimated the total amount of soil erosion during the baseline period 1950–1970 by considering sediment transport during fluvial network. The Yellow River is characteristic of high sediment delivery ratio (SDR) in the world [35], while most of world rivers show low SDR of 0.1–0.3 (refs 36-38). In comparison, numerous studies on sediment transport in the hilly regions of the Loess Plateau or to the Loess Plateau outlet, the Sanmenxia gauge station (Figure S1), show that its SDR is close to 1 with the SDR generally larger than 0.9 (refs 39,40). This indicates that more than 90% of the eroded sediment can be efficiently transported downstream while only <10% would be deposited within river channels. Based on an SDR of 0.9, the total amount of soil erosion was estimated to be 2680±520 Tg yr^{-1} for the baseline period (Table S3 below).

By using sedimentological surveys and statistical models, the eroded soils in the Yellow River basin before large-scale human interventions (i.e., the period prior to 1970) ranged from 2000 to 2500 Tg per year [41-44]. Thus, our estimate is consistent with previous soil erosion results. Using the same spatial interpolation method, the total amount of soil erosion during the period 2000–2015 was estimated at 1380±150 Tg yr^{-1}, indicating a 48.5% reduction from the baseline period. This value is close to recent model-based estimates of soil erosion rate on the Loess Plateau. By using the Universal Soil Loss Equation (USLE) model, for example, Fu et al. (2011) estimated the total soil loss on the entire Loess Plateau [45], 73% of which is located inside the Yellow River basin (Figure S1) and provides about 90% of the Yellow River sediment. Their estimates show that the potential soil loss on the Loess Plateau is 1510 Tg in 2008 (Figure S6) and the multi-year

average for the period 2000–2008 is 1990 Tg yr^{-1}. Furthermore, the average sediment flux at Sanmenxia Hydrological Gauge (Figure S1) prior to the operation of the Sanmenxia Dam (1960) is 1760 Tg yr^{-1} (Figure S7). Particularly, the average sediment flux for the highest four sediment loading years was 2580 Tg yr^{-1} (Figure S7). In consideration of the sediment deposition within the Yellow River main-stem channel (i.e., in the 3 sediment sinks in the upper, middle, and lower main-stem channel; ref 51), the reconstructed soil erosion is close to these gauge-based observations.

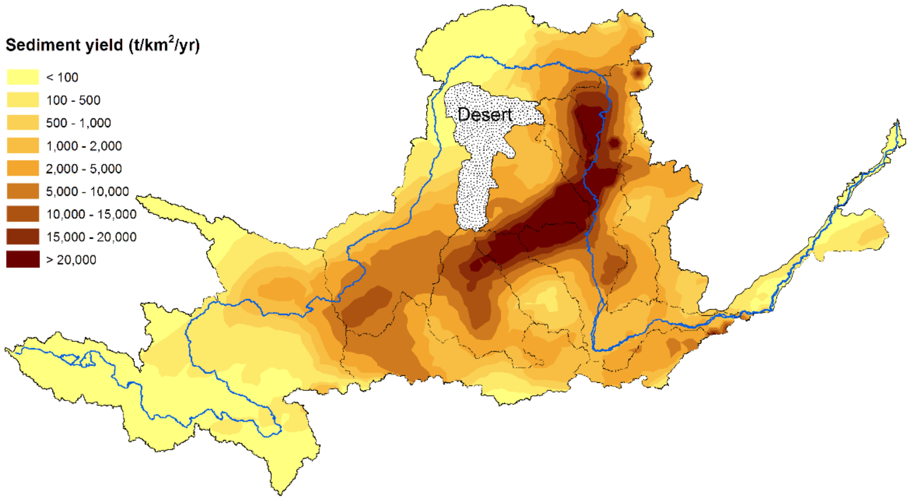

Figure S5. Spatial variability of sediment yield within the Yellow River basin. Adapted from Ran et al. (2013)(ref 34).

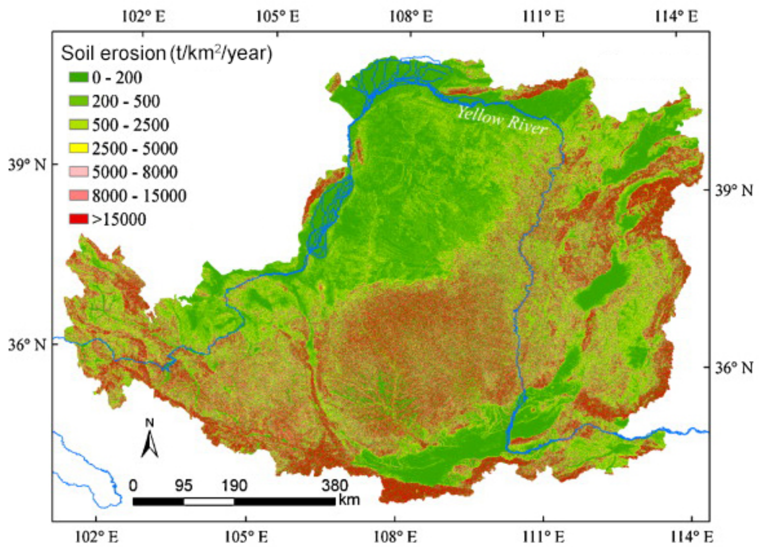

Figure S6. Spatial variability of potential soil erosion on the Loess Plateau in 2008. Adapted from Fu et al. (2011)(ref 45).

In comparison, Zhao et al. (2016) estimated the total soil erosion rate in the Yellow River basin in the 1950s by considering the sediment sources of topsoil erosion, gully erosion, and land-sliding separately [46]. Comparing their estimate with sediment fluxes at main-stem hydrological gauges suggests that they might have greatly underestimated the soil erosion rate. For example, their soil erosion estimates of 1270±430 Tg yr^{-1} for the 1950s is even lower than the observed

sediment flux at Sanmenxia Hydrological Gauge (1760±790; Figure S7) or Huayuankou Hydrological Gauge (1530±620; see its location in Figure S1) over the same time period [47,48]. Given the extremely high sediment delivery ratio (SDR) as discussed above and the minor contribution of bank erosion to fluvial sediment [4,47], the total soil erosion rate could be reasonably expected to be close to, or slightly higher than, the measured sediment fluxes at the Loess Plateau outlet (i.e., the Sanmenxia or Huayuankou gauges; Figure S1). Therefore, our estimated soil erosion amount in this study based on sediment yield map and a high SDR is reliable with high confidence, reflecting the strong soil erosion intensity and efficient sediment transport from hillslope eroding sources to the Yellow River main-stem channel.

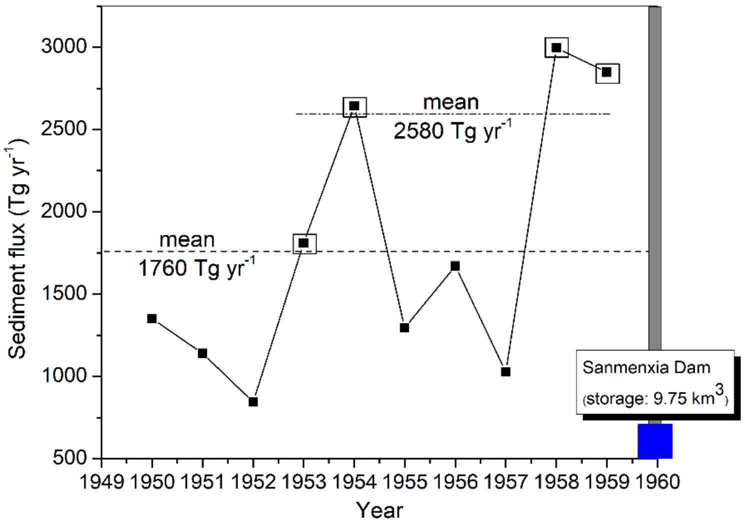

Figure S7. Temporal variation of sediment flux at Sanmenxia Hydrological Gauge during the period 1950–1959, before the operation of the Sanmenxia Dam in 1960. The points encircled by squares denote the highest four sediment loading years.

4. Assessment of SOC dynamics

Given that direct sampling results of SOC in the period 1950–1970 are not available, we used the second national soil survey results to quantify the amount of mobilized SOC. This national soil survey was conducted during the period 1979–1985 (ref. 49), and the spatial resolution of the compiled raster dataset is 1×1 km^2. Because progressive soil conservation has started since the early 1970s, it is recognized that using this SOC data set may have introduced bias to estimate erosion-induced SOC for the baseline period. However, given the considerably limited revegetation effect during this period [50], the resulting error is believed to be low. For the Yellow River basin, there are 9213 polygons with each polygon including one soil profile (0–100 cm deep). We divided the soil profiles into two depth classes, including the top soils (0–30 cm) and the subsurface soils (30–100 cm). The SOC content decreased with increasing depth, and the SOC in top soils was characterized by significant spatial variability. This spatial heterogeneity is particularly apparent between the headwater region and the middle reach (i.e., the Loess Plateau; Figure S1). The average SOC in top soils was 0.84±0.12% (ref. 51). The SOC content on the Loess Plateau was generally low, ranging from 0.2% to 0.8%, although high SOC contents (i.e., >1.5%) could be sporadically discovered in some local areas, usually on floodplains along river valleys. In comparison, the headwater regions showed a substantially higher SOC content due largely to the extensive development of alpine meadow and thus higher OC inputs from

meadow residues. While the SOC in most areas of the headwater region varied from 1.2% to 4.5%, the SOC content in the southmost part with widespread coverage of peatland can be as high as 39% with an areal average of 6–12% (ref 51).

The Yellow River sediment is characterized by a significant contribution from gully erosion which mobilizes large quantities of subsurface soils. More than 50% of the Yellow River sediment was originated from gully erosion [52], while the remaining fraction came from surface soils as a result of hillslope erosion. In addition, given that the Loess Plateau in the middle Yellow River basin provided 90% of the total sediment, we assumed 0.84% to be representative of the SOC content of the mobilized soils by water erosion. Because the light fraction of SOC would be preferentially mobilized during erosion processes, the eroded soils tend to be enriched in SOC relative to the parent soils [53]. For the Yellow River basin with exceptionally high erosion intensity and rapid water transport, its SOC enrichment ratio is typically low. Recent studies based on plot-scale measurements suggest that the enrichment ratio ranged from 0.8 to 1.2 (refs 53,54). Therefore, we used an enrichment ratio of 1.1 for the eroded top soils and 0.8 for the eroded subsurface soils. In association with the estimated soil erosion at the basin scale, the mobilized SOC during the baseline period 1950–1970 was estimated to be 21.4 ± 5.2 Tg C yr^{-1} (Table S3 below).

Reservoirs and dams play an important role in controlling soil erosion and trapping sediment in the Yellow River basin. Since the 1970s, more than 3100 reservoirs (Figure S8) have been constructed [1,55]. In addition, silt check dams are an important and effective strategy in intercepting sediment and preventing sediment from being transported downstream into main-stem river channels. Approximately, 1.07×10^5 silt check dams have been completed by 2015, including ~16,000 middle- and large-sized dams and 91,000 small-sized dams [56,57]. While most of the middle- and large-sized dams were built in the 1970s and the 2000s (ref 56), constructions of small dams were persistently carried out through the whole study period. Numerous more silt check dams are under construction and being planned, and in total 1.633×10^5 silt check dams will be put into operation in recent years [58]. These built reservoirs and silt check dams are mostly located on the Loess Plateau which had the highest soil erosion rates [55,56].

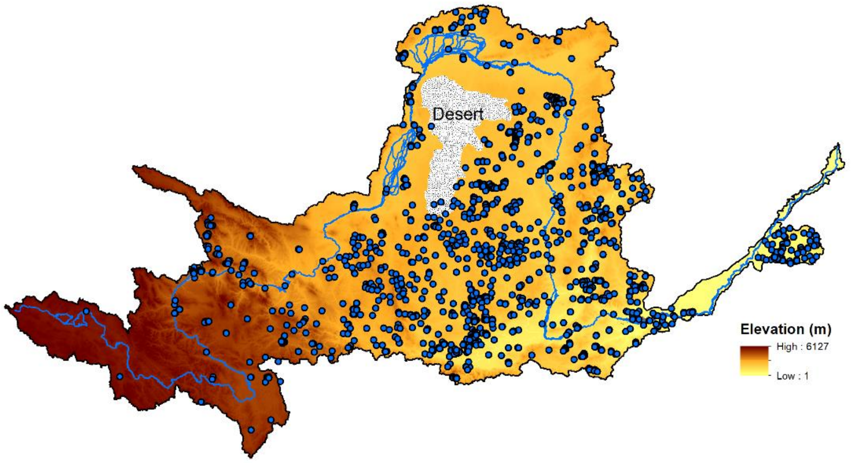

Figure S8. Spatial distribution of the constructed reservoirs within the Yellow River basin with a large proportion of reservoirs on the Loess Plateau. Refer to Figure S1 for the boundary of the Loess Plateau.

Based on sediment trapping efficiency of individual reservoirs and sediment input from their respective upstream catchment, we estimated the cumulative sediment trapping within the watershed. Reservoir sediment trapping is positively correlated with reservoir storage and spatial closeness to sediment yielding areas. Large reservoirs on the main-stem channel can generally trap more sediment, and those reservoirs located shortly downstream of the high sediment yielding areas would be rapidly filled up due to abundant sediment supply. The average sediment trapping by reservoirs was 590 Tg yr^{-1} during the period 2000–2015 (ref 34). In contrast to reservoirs that usually have multiple purposes, silt check dams are constructed solely for sediment trapping as engineering measures in soil conservation strategy. As a result of the high sediment deposition rate, the life time for silt check dams is usually short, less than 20 years or even shorter for those located in strong soil erosion regions [59,60]. A preliminary estimate indicates that more than 21,000 Tg of sediment has been trapped behind silt check dams on the Loess Plateau, which translates into an average sedimentation rate of 350 Tg yr^{-1} (ref 1). Due to the rapid sediment deposition and resulting abandonment of silt check dams, new dams are constantly constructed to replace the abandoned ones (Figure S9). The filled dams are generally reclaimed as croplands, usually for wheat and corn plantations. However, it is worth noting the spatial heterogeneity of sediment deposition in such a large river basin. Estimating the total sediment deposition by reservoirs or silt check dams is associated with great uncertainty, as is the buried OC, although the deposited sediments show a stable and low SOC content over space and time. Thus, the buried sediment and OC results are conservative with medium confidence.

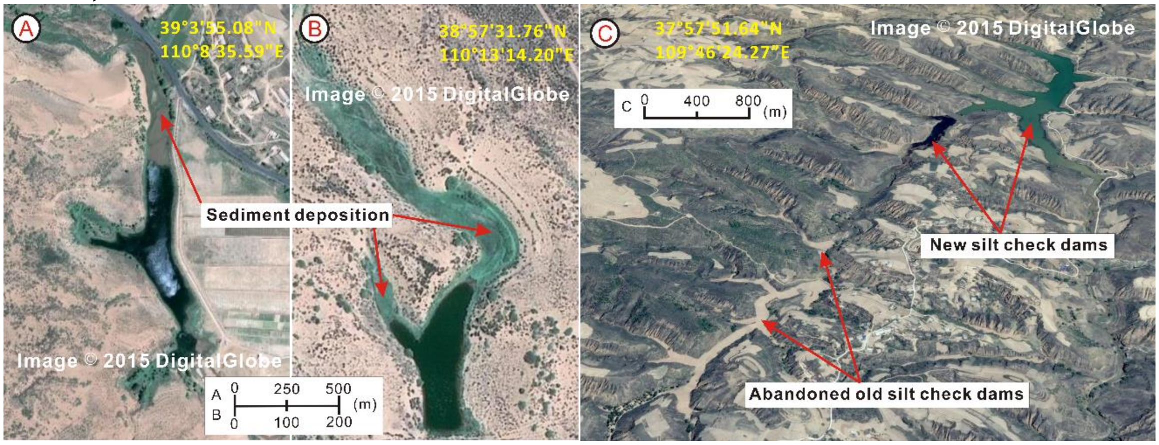

Figure S9. Typical examples of rapid sediment deposition behind silt check dams on the Loess Plateau (a and b), causing continuous silt check dam abandonments (c). The satellite images were obtained from Google Earth Pro (https://www.google.com/earth, map data: Google, DigitalGlobe) and the maps were created by using CorelDRAW (version: X6, https://www.coreldraw.com) by the first author (L Ran).

Sediment deposition within river channels during transport is another important sediment sink for large river basins, such as the Yellow River. Despite the exceptionally high SDR as a whole, there are three major sediment deposition zones along the Yellow River main-stem channel, located separately in the upper, middle, and lower reaches [51]. We quantified the sediment deposition with these sink zones on the basis of sediment load data measured at the input and output gauge stations for each zone with medium-to-high confidence. The stored sediment during the period 2000–2015 was 60±90 Tg yr^{-1}, and the associated OC was 0.3±0.5 Tg C yr^{-1} based on our recent particulate OC analysis [61]. Although there was no large-scale water withdrawal for agriculture and industry before 1970, recent decades have seen abrupt increases

in water withdrawal due largely to expanding economy (Figure 1 in the manuscript). Based on daily water diversion and suspended sediment concentration measured, the diverted sediment load was estimated at 90±19 Tg yr^{-1} during the period 2000–2015. Accordingly, 0.5±0.3 Tg of OC was annually diverted, mainly from the main-stem channel, based on our earlier OC analysis along the Yellow River main-stem channel [61]. Both fluxes were estimated with high confidence. Finally, the remaining sediment and OC terms (i.e., inland burial and hillslope redistribution for sediment, and decomposition for OC) were determined and their respective propagated uncertainty was determined by using the budgetary equations (Equations 1 and 2 in the manuscript).

5. Fluxes into the Bohai Sea

Sediment and OC transport from the Yellow River basin into the Bohai Sea were calculated from high-frequency sampling results at the lowermost Lijin gauge station on the Yellow River main-stem channel (Figure S1). The Lijin gauge station is located 104 km upstream of the river mouth and free of tidal influences, and it controls 99% of the drainage basin. Starting from 1934, daily flow discharge and sediment concentration were measured at the Lijin gauge station. The sampling procedures follow national standards of hydrological measurement formulated by the Ministry of Water Resources, China [62]. Because the OC transport in the reference scenario (1950–1970) is not available, we instead used the oldest sampling results measured in the early 1980s to estimate the OC flux into the Bohai Sea. This may have caused considerable errors to the actual OC flux of the 1950–1970 scenario. Therefore, we estimated it with medium confidence only while a high confidence for the sediment flux. For the annual OC transport in the period 2000–2015, we used our recent weekly sampling results during the period 2008–2012 (ref 61) for the calculation. Because the concentrations of OC (dissolved and particulate) were measured on a weekly basis whereas flow and sediment were continuously (daily) measured, the annual OC flux was calculated by using the Beale's stratified ratio estimator [63]. Given the long sequence of the measurements and the high sampling frequency, we estimated the annual sediment and OC fluxes for the period 2000–2015 with high confidence.

6. Uncertainty with SDR-based estimates

Assessments of the human-induced OC stabilization were based on budgetary results for the two scenarios (1950–1970 and 2000–2015, respectively). Therefore, quantifying the amount of the eroded soil within the basin was critical for accurately assessing the fate of the mobilized SOC and the impacts of soil conservation efforts. While we used the SDR of 0.9 representative of the Yellow River to estimate its soil erosion rate with high confidence, we also evaluated the possible errors by assuming lower SDR values that are characteristics of other rivers in the world (Figure S10). The decomposed OC increased from 8.8 to 87.1 Tg C yr^{-1} with the SDR decreasing from 0.9 to 0.1, and the magnitude of soil conservation in inducing a C sink may be greatly reduced.

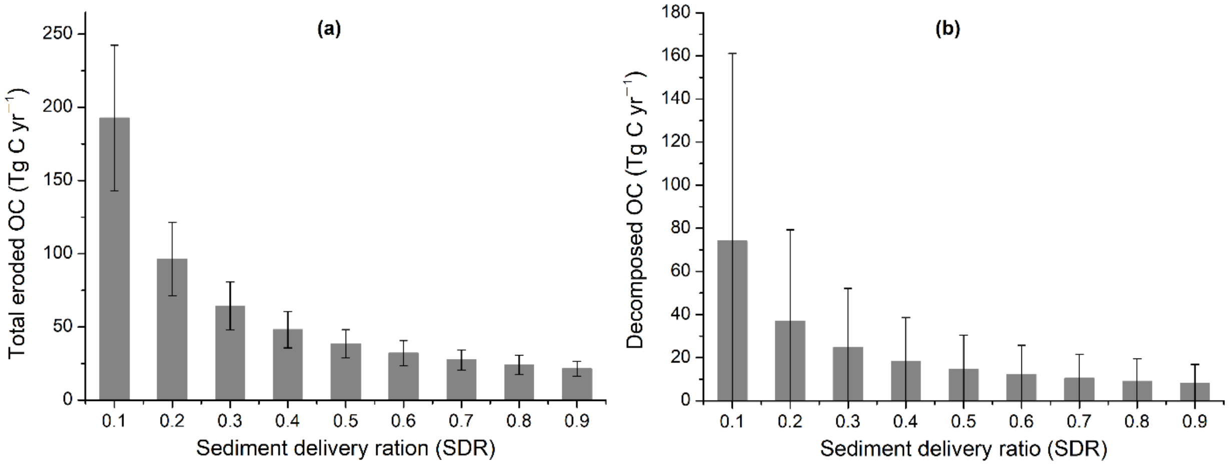

Figure S10. Great differences in total eroded OC (a) and decomposed OC induced by erosion (b) under different sediment delivery ratio (SDR) scenarios. Range bars show associated uncertainties for each SDR scenario. As a means of quantifying sediment deposition and storage, SDR is defined as the ratio of sediment delivered at the catchment outlet to gross soil erosion within a given river basin [36].

7. Presentation of OC budgetary results

Derived from the erosion, transport, and deposition processes of erosion-induced OC, the quantified terms of the budget equations (see Methods in the manuscript) for the baseline scenario (1950–1970) and the period after the implementation of the Grain-for-Green Project (2000–2015) are presented in Table S3. The results are reported as mean ± standard deviation. In addition, a brief description on the estimation method and the accuracy and confidence for each term are also provided.

Table S3. Budgetary results of the erosion-induced OC transport within the Yellow River basin (mean ± standard deviation).

Term	Sediment[a]	OC content	OC flux	Brief description
	Tg yr^{-1}	%	Tg C yr^{-1}	
**Baseline scenario (1950–1970)**				
Soil erosion	2680±520	0.84±0.12	21.4±5.2[b]	Soil erosion is estimated from sediment yield map generated from 179 gauge stations and a high SDR value (i.e., 0.9) (refs 3,35,40,64). High confidence for erosion estimate. The OC content is based on the second national soil survey results since 1979 (ref 51). This may have slightly overestimated the OC value.
Seaward transport	1200±530	0.51±0.28	6.1±4.3	Average of the sediment fluxes at Lijin gauge (Figure S1) based on daily data. Because the OC is highly stable over space and time[65], it is based on high-frequency sampling results in the 1980s (refs 66-68). High confidence for sediment flux and medium confidence for OC flux.
Inland burial	1660±700	0.49±0.29	7.3±5.7	Determined as a residual between soil erosion and seaward sediment transport flux.
Decomposition	/	/	8.0±8.8	Determined as a residual and includes the propagated uncertainty.
**2000–2015 scenario**				
Soil erosion	1380±150	0.85±0.30	11.1±4.1[b]	Based on the sum of all sediment transport terms and a SDR of 0.9. The OC data are from recent literature [11,19,69-71]. High confidence for both estimates.
Seaward transport	126±100	0.54±0.27	0.7±0.6	Average of the sediment fluxes at Lijin gauge based on daily data. OC data is from recent high-frequency sampling at Lijin gauge [61]. High confidence for both fluxes.
Dam trapping	940±30	0.35±0.16	3.3±1.5	Sum of reservoir and silt check dam trapping [34]. We estimate OC by considering reservoirs and silt check dams separately [51]. Medium confidence for both fluxes.
Channel sedimentation	60±90	0.53±0.23	0.3±0.5	Based on sedimentation in three sediment sink zones[c]. Medium-to-high confidence for sediment estimate. The OC data are from our recent sampling at Toudaoguai, Tongguan, and Lijin stations (Figure S1) [61]. High confidence for OC content.
Sediment diversion	90±10	0.52±0.26	0.5±0.3	Based on the volume of diverted water from main-stem and its sediment content. The OC data are from sampling near the irrigation regions, i.e., at Toudaoguai [61]. High confidence for both the sediment and OC terms.
Hillslope redistribution	160±200	0.85±0.28	1.0±1.3	Determined as a residual and includes the propagated uncertainty for the sediment.
Decomposition	/	/	5.3±4.6	Determined as a residual and includes the propagated uncertainty.
NPP accumulation			4.3±0.9	Estimated from the version-55 of the Terra/MODIS NPP products (MOD 17A3). Medium-to-high confidence with accuracy of <9% (NASA LP DAAC, 2016).
SOC stock increment			2.7±0.9	Based on 1366 soil profiles from 21 studies conducted on the Loess Plateau (medium-to-high confidence). All soil profiles were drilled after the introduction of the Grain-for-Green Project (i.e., since 2000).

[a] It refers to suspended sediment because the bedload fraction is difficult to determine and is very small in quantity for the Yellow River (i.e., <1%) (refs 5,34). Seaward sediment flux is collected from the Yellow River Conservancy Commission: http://www.yellowriver.gov.cn/nishagonggao/. [b] OC enrichment for the eroded soils was considered. The enrichment ratio for the OC from topsoils and gully erosion was assumed to 1.1 and 0.8, respectively, given that gully erosion contributes to ~50% of the total eroded soils [35]. [c] Locations of the three sediment sink zones can be found in Ran et al. (2014) (ref 51).

References

1 Xin, Z., Ran, L. & Lu, X. Soil erosion control and sediment load reduction in the Loess Plateau: Policy perspectives. *International Journal of Water Resources Development* **28**, 325-341 (2012).

2 Hassan, M. A., Church, M., Xu, J. & Yan, Y. Spatial and temporal variation of sediment yield in the landscape: Example of Huanghe (Yellow River). *Geophys Res Lett* **35**, L06401, doi:10.1029/2008gl033428 (2008).

3 Walling, D. E. & Fang, D. Recent trends in the suspended sediment loads of the world's rivers. *Global and Planetary Change* **39**, 111-126 (2003).

4 Wang, H. J. *et al.* Stepwise decreases of the Huanghe (Yellow River) sediment load (1950-2005): Impacts of climate change and human activities. *Global and Planetary Change* **57**, 331-354 (2007).

5 Wu, B., Wang, G., Xia, J., Fu, X. & Zhang, Y. Response of bankfull discharge to discharge and sediment load in the Lower Yellow River. *Geomorphology* **100**, 366-376 (2008).

6 Li, C., Yang, Z. & Wang, X. Trends of annual natural runoff in the Yellow River Basin. *Water international* **29**, 447-454 (2004).

7 Yellow River Conservancy Commission. Yellow River Sediment Bulletin, (http://www.yellowriver.gov.cn/nishagonggao/). Access: 25 April 2016 (2016).

8 NASA LP DAAC. Terra/MODIS Net Primary Production Yearly L4 Global 1km. (https://lpdaac.usgs.gov/dataset_discovery/modis/modis_products_table/mod17a13). Access: 18 August 2016 (2016).

9 Liu, Z., Shao, M. a. & Wang, Y. Effect of environmental factors on regional soil organic carbon stocks across the Loess Plateau region, China. *Agriculture, Ecosystems & Environment* **142**, 184-194 (2011).

10 Wang, Y., Fu, B., Lü, Y. & Chen, L. Effects of vegetation restoration on soil organic carbon sequestration at multiple scales in semi-arid Loess Plateau, China. *CATENA* **85**, 58-66 (2011).

11 Chen, L. *et al.* Effect of land use conversion on soil organic carbon sequestration in the loess hilly area, loess plateau of China. *Ecol Res* **22**, 641-648 (2007).

12 Chang, X. *et al.* Soil organic carbon accumulation in abandoned croplands on the Loess Plateau. *Land Degradation & Development* **28**, 1519–1527 (2017).

13 Xin, Z., Qin, Y. & Yu, X. Spatial variability in soil organic carbon and its influencing factors in a hilly watershed of the Loess Plateau, China. *Catena* **137**, 660-669 (2016).

14 Zhao, B. *et al.* Spatial distribution of soil organic carbon and its influencing factors under the condition of ecological construction in a hilly-gully watershed of the Loess Plateau, China. *Geoderma* **296**, 10-17 (2017).

15 Deng, L., Wang, G., Liu, G. & Shangguan, Z. Effects of age and land-use changes on soil carbon and nitrogen sequestrations following cropland abandonment on the Loess Plateau, China. *Ecological Engineering* **90**, 105-112 (2016).

16 Shi, S. & Han, P. Estimating the soil carbon sequestration potential of China's Grain for Green Project. *Global Biogeochemical Cycles* **28**, 1279-1294 (2014).

17 Chen, Y. *et al.* Balancing green and grain trade. *Nat Geosci* **8**, 739-741 (2015).

18 Fu, X., Shao, M., Wei, X. & Horton, R. Soil organic carbon and total nitrogen as affected by vegetation types in Northern Loess Plateau of China. *Geoderma* **155**, 31-35 (2010).

19 Wang, Y., Fu, B., Lü, Y., Song, C. & Luan, Y. Local-scale spatial variability of soil organic carbon and its stock in the hilly area of the Loess Plateau, China. *Quaternary Research* **73**, 70-76 (2010).
20 Wang, Z. *et al.* Temporal and spatial variations in soil organic carbon sequestration following revegetation in the hilly Loess Plateau, China. *CATENA* **99**, 26-33 (2012).
21 Chen, H. *et al.* Effects of 11 years of conservation tillage on soil organic matter fractions in wheat monoculture in Loess Plateau of China. *Soil and Tillage Research* **106**, 85-94 (2009).
22 Zhao, W. *et al.* Effect of different vegetation cover on the vertical distribution of soil organic and inorganic carbon in the Zhifanggou Watershed on the loess plateau. *Catena* **139**, 191-198 (2016).
23 Chang, R., Fu, B., Liu, G., Wang, S. & Yao, X. The effects of afforestation on soil organic and inorganic carbon: A case study of the Loess Plateau of China. *Catena* **95**, 145-152 (2012).
24 Deng, L., Wang, K.-B., Chen, M.-L., Shangguan, Z.-P. & Sweeney, S. Soil organic carbon storage capacity positively related to forest succession on the Loess Plateau, China. *Catena* **110**, 1-7 (2013).
25 Jin, Z. *et al.* Natural vegetation restoration is more beneficial to soil surface organic and inorganic carbon sequestration than tree plantation on the Loess Plateau of China. *Sci Total Environ* **485**, 615-623 (2014).
26 Cheng, M., Xue, Z., Xiang, Y., Darboux, F. & An, S. Soil organic carbon sequestration in relation to revegetation on the Loess Plateau, China. *Plant and soil* **397**, 31-42 (2015).
27 Li, Z. *et al.* Response of soil organic carbon and nitrogen stocks to soil erosion and land use types in the Loess hilly–gully region of China. *Soil and Tillage Research* **166**, 1-9 (2017).
28 Han, X. *et al.* Soil carbon and nitrogen sequestration under several different forest lands converted by farmland in Loess Hilly Area. *Journal of Agro-Environment Science* **31** (2012).
29 Fang, X., Xue, Z., Li, B. & An, S. Soil organic carbon distribution in relation to land use and its storage in a small watershed of the Loess Plateau, China. *Catena* **88**, 6-13 (2012).
30 Zhao, X., Wu, P., Gao, X. & Persaud, N. Soil quality indicators in relation to land use and topography in a small catchment on the Loess Plateau of China. *Land Degradation & Development* **26**, 54-61 (2015).
31 Wu, X., Zhang, X., Xie, Y., Xu, K. & Yang, J. Vertical distribution characters of soil organic carbon and soil enzyme activity in alfalfa field with different growing years. *Acta Prataculturae Sinica* **22**, 245-251 (in Chinese) (2013).
32 Zhao, X., Li, J. & Li, H. Effects of vegetation restoration type on soil carbon,nitrogen,and microbial quantity in Guandi Mountain. *Chinese Journal of Ecology* **39**, 2102-2110 (in Chinese) (2010).
33 Wang, L., Zhang, Q., Niu, X., Yang, Z. & Zhang, J. Effects of different land-uses on soil physical and chemical properties in the Loess Plateau of Shanxi Province. *Chinese Journal of Eco-Agriculture* **15**, 53-56 (in Chinese) (2007).
34 Ran, L., Lu., X. X., Xin, Z. B. & Yang, X. Cumulative sediment trapping by reservoirs in large river basins: A case study of the Yellow River basin. *Global and Planetary Change* **100**, 308-319 (2013).
35 Xu, J. Erosion caused by hyperconcentrated flow on the Loess Plateau of China. *CATENA* **36**, 1-19 (1999).
36 Walling, D. The sediment delivery problem. *Journal of Hydrology* **65**, 209-237 (1983).

37 Syvitski, J. P. & Milliman, J. D. Geology, geography, and humans battle for dominance over the delivery of fluvial sediment to the coastal ocean. *The Journal of Geology* **115**, 1-19 (2007).
38 De Vente, J., Poesen, J., Arabkhedri, M. & Verstraeten, G. The sediment delivery problem revisited. *Progress in Physical Geography* **31**, 155-178 (2007).
39 Jing, K. A study on gully erosion on the Loess Plateau. *Scientia Geographica Sinica* **6**, 340-347 (in Chinese) (1986).
40 Zhao, G., Mu, X., Wen, Z., Wang, F. & Gao, P. Soil erosion, conservation, and eco-environment changes in the Loess Plateau of China. *Land Degradation & Development* **24**, 499-510 (2013).
41 Chen, Y. A preliminary analysis of the processes of sediment yield in small catchment on the Loess Plateau. *Geographical Research* **2**, 35-47 (in Chinese) (1983).
42 Fu, B. Soil erosion and its control in the loess plateau of China. *Soil Use and Management* **5**, 76-82 (1989).
43 Shi, D. Effects of soil erosion on ecological environment and natural disasters. *Advances in Earth Science* **4**, 41-44 (in Chinese) (1990).
44 Wang, F., Li, R. & Yang, Q. Review on effects of human activities on the soil erosion in the Loess Plateau. *Journal of Sediment Research* **5**, 74-80 (in Chinese) (2003).
45 Fu, B. *et al.* Assessing the soil erosion control service of ecosystems change in the Loess Plateau of China. *Ecological Complexity* **8**, 284-293 (2011).
46 Zhao, J., Oost, K. V., Chen, L. & Govers, G. Moderate topsoil erosion rates constrain the magnitude of the erosion-induced carbon sink and agricultural productivity losses on the Chinese Loess Plateau. *Biogeosciences* **13**, 4735-4750 (2016).
47 Peng, J., Chen, S. & Dong, P. Temporal variation of sediment load in the Yellow River basin, China, and its impacts on the lower reaches and the river delta. *CATENA* **83**, 135-147 (2010).
48 Wang, S., Fu, B., Liang, W., Liu, Y. & Wang, Y. Driving forces of changes in the water and sediment relationship in the Yellow River. *Sci Total Environ* **576**, 453-461 (2017).
49 Yang, Y., Mohammat, A., Feng, J., Zhou, R. & Fang, J. Storage, patterns and environmental controls of soil organic carbon in China. *Biogeochemistry* **84**, 131-141 (2007).
50 Wang, S. *et al.* Reduced sediment transport in the Yellow River due to anthropogenic changes. *Nat Geosci* **9**, 38-41 (2015).
51 Ran, L., Lu, X. X. & Xin, Z. B. Erosion-induced massive organic carbon burial and carbon emission in the Yellow River basin, China. *Biogeosciences* **11**, 945-959 (2014).
52 Xu, J. A study of physico-geographical factors for formation of hyperconcentrated flows in the Loess Plateau of China. *Geomorphology* **24**, 245-255 (1998).
53 Quinton, J. N., Govers, G., Van Oost, K. & Bardgett, R. D. The impact of agricultural soil erosion on biogeochemical cycling. *Nature Geosci* **3**, 311-314 (2010).
54 Wang, Y., Zhang, X. & Han, F. Profile variability of soil properties in check dams on the Loess Plateau and its functions. *Environmental Science* **29**, 1020-1026 (in Chinese) (2008).
55 Ran, L. & Lu, X. X. Delineation of reservoirs using remote sensing and their storage estimate: an example of the Yellow River basin, China. *Hydrological Processes* **26**, 1215-1229 (2012).
56 Liu, X., Gao, Y. & Wang, F. Quantity and distribution of silt check dams that still have sediment retaining ability in the Loess Plateau. *Yellow River* **39**, 1-5 (in Chinese) (2017).
57 Qu, C., Liu, W., Liu, C., Wang, T. & Jia, J. Research progress on soil-retaining dam in Loess Plateau. *Bulletin of Soil and Water Conservation* **36**, 339-342 (in Chinese) (2016).

58 Zhang, H. *et al.* Loess Plateau check dams can potentially sequester eroded soil organic carbon. *Journal of Geophysical Research: Biogeosciences* **121**, 1449-1455 (2016).
59 Zhao, Y., Mu, X., Yan, B. & Zhao, G. Influence of vegetation restoration on runoff and sediment of Yanhe basin. *Journal of Sediment Research* **4**, 67-73 (in Chinese) (2014).
60 Xu, Y., Fu, B. & He, C. Assessing the hydrological effect of the check dams in the Loess Plateau, China, by model simulations. *Hydrol Earth Syst Sc* **17**, 2185-2193 (2013).
61 Ran, L. *et al.* Spatial and seasonal variability of organic carbon transport in the Yellow River, China. *Journal of Hydrology* **498**, 76-88 (2013).
62 Bai, Z., Xu, Y.-P., Hu, J. & Huang, Y. Verification of Interpolated Variance Estimate Method for Discharge Measurements in Yangzte River Basin, China. *Flow Measurement and Instrumentation* **60**, 155-163 (2018).
63 Dolan, D. M., Yui, A. K. & Geist, R. D. Evaluation of river load estimation methods for total phosphorus. *Journal of Great Lakes Research* **7**, 207-214 (1981).
64 Jing, K., Chen, Y. & Li, F. *Sediment and Environment of Yellow River*. (Science Press, 1993).
65 Liu, D. & Zhang, L. Temporal and spatial distributions of organic carbon in the Huanghe (Yellow) River. *Periodical of Ocean Univeristy of China* **40**, 105-110 (in Chinese) (2010).
66 Cauwet, G. & Mackenzie, F. T. Carbon inputs and distribution in estuaries of turbid rivers: the Yang Tze and Yellow rivers (China). *Marine Chemistry* **43**, 235-246 (1993).
67 Cai, D. Geochemical studies on organic carbon isotope of the Huanghe River (Yellow River) estuary. *Sci China Ser B* **37**, 1001-1015 (1994).
68 Zhang, S., Gan, W.-B. & Ittekkot, V. Organic matter in large turbid rivers: the Huanghe and its estuary. *Marine Chemistry* **38**, 53-68 (1992).
69 Liu, S., Guo, S., Wang, X. & Xue, B. Effect of vegetation on soil organic carbon of slope land in gully region of Loess Plateau. *Journal of Natural Resources* **20**, 529-536 (in Chinese) (2005).
70 Li, L., Tu, C., Zhao, Z., Cui, L. & Liu, W. Distribution characteristics of soil organic carbon and its isotopic composition for soil profiles of Loess Plateau under different vegetation condictions. *Earth and Environment* **39**, 441-449 (in Chinese) (2011).
71 Jia, S., He, X., Chen, Y. & Zheng, F. Effect of soil erosion on soil organic carbon loss on the loess hilly areas. *Research of Soil and Water Conservation* **11**, 88-90 (in Chinese) (2004).
